# Simulation as a key training method for inculcating public health leadership skills: a mixed methods study

**DOI:** 10.3389/fpubh.2023.1202598

**Published:** 2023-07-06

**Authors:** Keren Dopelt, Itamar Shevach, Ofek Eliad Vardimon, Katarzyna Czabanowska, Jascha De Nooijer, Robert Otok, Lore Leighton, Osnat Bashkin, Mariusz Duplaga, Hagai Levine, Fiona MacLeod, Maureen Malowany, Leah Okenwa-Emegwa, Shira Zelber-Sagi, Nadav Davidovitch, Paul Barach

**Affiliations:** ^1^Department of Health Policy and Management, School of Public Health, Faculty of Health Sciences, Ben Gurion University of the Negev, Beer Sheva, Israel; ^2^Department of Public Health, Ashkelon Academic College, Ashkelon, Israel; ^3^Department of International Health, Care and Public Health Research Institute CAPHRI, Faculty of Health, Medicine and Life Sciences, Maastricht University, Maastricht, Netherlands; ^4^Department of Health Promotion, School of Health Professions Education, Faculty of Health, Medicine and Life Sciences, Maastricht University, Maastricht, Netherlands; ^5^The Association of Schools of Public Health in the European Region (ASPHER), Brussels, Belgium; ^6^Department of Health Promotion and e-Health, Institute of Public Health, Faculty of Health Sciences, Jagiellonian University Medical College, Krakow, Poland; ^7^The Israeli Association of Public Health Physicians (IPAPH), Israeli Medical Association, Ramat-Gan, Israel; ^8^Braun School of Public Health and Community Medicine, Faculty of Medicine, Hebrew University of Jerusalem-Hadassah, Jerusalem, Israel; ^9^School of Public Health, University College Cork, Cork, Ireland; ^10^Department of Health Sciences, The Swedish Red Cross University (SRCU), Huddinge, Sweden; ^11^School of Public Health, Faculty of Social Welfare and Health Sciences, University of Haifa, Haifa, Israel; ^12^College of Population Health, Thomas Jefferson University, Philadelphia, PA, United States; ^13^Interdisciplinary Research Institute for Health Law and Science, Sigmund Freud University Vienna, Vienna, Austria; ^14^Department of Surgery, Imperial College School of Medicine, London, United Kingdom

**Keywords:** simulation, leadership, communication skills, Kolb’s experimental learning, ethical dilemma

## Abstract

**Background:**

Successful management of public health challenges requires developing and nurturing leadership competencies. We aimed to evaluate the effectiveness of training simulations to assess public health leadership and decision-making competencies during emergencies as an effective learning and training method.

**Methods:**

We examined the effects of two simulation scenarios on public health school students in terms of their experience (compared to face-to-face learning) and new skills acquired for dealing with similar emergent situations in the future. A mixed-methods design included developing a validated and pre-tested questionnaire with open-and closed-ended questions that examined the simulation impact and the degree of student satisfaction with the conditions in which it was conducted. Semi-structured in-depth interviews were conducted with the students after going through the simulations. The questionnaire results were evaluated using descriptive analytics. The interviews were analyzed using thematic analyses. All data were collected during June 2022.

**Results:**

The questionnaire results indicate that students strengthened their interpersonal communication skills and learned about the importance of listening to the opinions of others before formulating their positions. Four themes emerged from 16 in-depth interviews, according to Kolb’s experimental learning cycle. Students emphasized the effectiveness of experiential learning versus traditional classroom learning. The simulation scenarios were felt to realistically convey critical issues regarding leadership, decision-making, and teamwork challenges. They effectively conveyed the importance of building a culture of conducting substantive and respectful discussions.

**Conclusion:**

Simulation is a powerful pedagogical training tool for public health leadership competencies. Simulations were seen to be advantageous over face-to-face learning in imparting a range of leadership skills and hands-on practice. We recommend integrating simulations in all public health leadership training programs.

## 1. Introduction

Successful management of public health emergencies such as dealing with pandemics, earthquakes, fires, and other natural and man-made disasters, require the development and deployment of leadership competencies (1). Simulation during the COVID-19 pandemic helped refine protocols, facilitate practice changes, uncover safety gaps, improve response to crisis situations, supported team training and systems integration, and train redeployed healthcare workers in unfamiliar roles (2). Simulation helps train for essential clinical and leadership competencies using experiential learning supporting the quadruple aim (3).

Learning is the acquired behavior or potential change resulting from instruction, training, and practice or experience (4). In the context of professional training at a graduate level, learning is goal oriented and motivated by progress toward independent practice. Simulation is an effective, evidence-based learning tool that supports experiential learning and improves critical thinking (5, 6). Simulation has been shown to support the rapid acquisition of multiple skills, such as clinical skills, therapeutic procedures, time management, teamwork, and decision-making under pressure (7). Simulation based on Kolb’s Experimental Learning Cycle (8) supports learners benefiting from a direct and experiential encounter with a significant phenomenon, the investigation of which requires reflective observations of knowledge and experience. Kolb’s experiential learning model posits that learning includes four stages that repeat themselves: Concrete Experience, Reflective Observation, Abstract Conceptualization, and Active Experimentation, and that occur through a cycle of reflective observations of concrete experiences to gain a deeper understanding of what can be learned from each experience (9). New ideas are then applied to future experiences, renewing the cycle. Unlike traditional learning, Kolb’s model enables knowledge acquired in one experience to be applied to other, new, and unfamiliar experiences. The process of reflective observations and building perceptions provides tools and supports skills that allow an encounter with a new and unfamiliar situation to succeed under ambiguity and uncertainty (10).

Simulations for the development of leadership skills are applied worldwide in all health professions and at all career stages and have been proven to be effective in developing management and leadership skills (for example, (11–14)). The skills identified as significant for healthcare leaders include communication skills, teamwork, problem and conflict resolution, interpersonal skills, ability to work under pressure, negotiation ability, ability to motivate people, and entrepreneurship (15, 16). Simulation experiences can help clinical teams strengthen their leadership self-confidence when performing professional work in fulfilling their roles as public servants (17). Simulations can effectively replicate conflict situations and strengthen communication skills and provide effective ways to resolve conflicts. They allow health system workers to translate theoretical learning into practice (18), improve communication skills (19), physician learners to improve patient handovers and develop ethical behavioral norms that support management of leadership dilemmas (20).

Hertelendy et al. (21) found that 55% of accredited Master of Public Health programs in the United States provide Leadership training courses, and only one program offered a crisis resource management leadership course. The authors state that the COVID-19 pandemic and climate change brought emergencies to the forefront for health systems, so public health curricula must emphasize leadership competencies to prepare their graduates to lead complex crisis events.

We developed and evaluated the effectiveness of simulation training of public health leadership and decision-making competencies during periods of emergency as a pedagogical and learning method. The research examined the effects of the simulation training on participants in terms of their experience (compared to traditional frontal learning), the strengthening of their skills and the new competencies acquired for dealing with similar emergent situations in the future.

## 2. Materials and methods

### 2.1. Orientation to the study

This prospective, mixed-methods study is part of a larger multinational Erasmus Plus funded project for building Capacity in Higher Education entitled “Sharing European Educational Experience in Public Health for Israel (SEEEPHI): harmonization, employability, leadership, and outreach,” described in Bashkin et al. (22). Initial findings from the project point to considerable gaps between the needs of the public health labor force employers and the curriculum of Israeli Schools of Public Health, indicating a paucity of leadership training (23, 24).

### 2.2. Setting and participants

We developed and piloted a leadership course using training simulations designed for health professions in a recently opened learning track for healthcare management as part of a Master degree in Health Policy and Management at the Ben Gurion University School of Public Health (KD and ND academic coordinators of the program). The simulations were aimed at improving the ability to manage conflicts, strengthen teamwork, encourage collaborations with parties outside the healthcare system, and motivate a multi-disciplinary team. Eighteen students took the course and participated in the study at Ben Gurion University. The students were divided into three facilitated groups of six students (led by ND, KD, and IS).

### 2.3. Procedure

#### 2.3.1. Simulation

The students were given general instructions about the training simulations followed by extensive debriefing, conducted on Friday, June 3, 2022, at the Faculty of Health Sciences Simulation Center at Ben Gurion University (Appendix 1). Each group was assigned a room with a small table, chairs, and a blackboard. Each group’s facilitator provided participants with their specific role description. Students were instructed to act according to their assigned role. The simulations were filmed. The students participated in the two simulations described in Appendix 1. The questionnaires (Appendix 2), requiring about 10 min to complete, were distributed to all participants after each simulation. The simulation videos were used for debriefing by the facilitator together with the participants.

#### 2.3.2. Structured self-reported questionnaire

We developed and piloted a questionnaire (Appendix 2) that included 22 questions, including four questions on demographics (profession, role in the simulation, age, and whether they participated in a simulation in the past), nine open-ended questions, and nine closed-ended questions using a Likert scale from 1 to 5. The open-ended questions explored whether the students had the knowledge and skills to meet the learning goals of the scenario, what gaps they identified in their knowledge base and preparation for the simulation. The closed-ended questions examined the degree of satisfaction with the conditions in which the simulation was conducted, how confident they felt in managing the situation, the contribution of the simulation to their sense of security and confidence. Ten nursing students at Ben Gurion University who participated in previous simulations pretested the questionnaire, and the final version was modified to address their feedback. All participants completed the questionnaire anonymously.

#### 2.3.3. The interview

We interviewed 16 students (out of the 18 participants) in June 2022, during the 2 weeks following their participation in the simulations, using a pretested semi-structured individual interview tool based on our interview guides (Appendix 3). Two students were abroad at the time of the interviews and were not interviewed. The interviews were conducted in Hebrew (by IS, a lawyer with an MBA in business administration and an MA in Emergency Medicine), trained to conduct the interviews by two highly experienced qualitative researchers (by KD and ND). Two pilot joint interviews were conducted for demonstration and practice. Before the interview, each interviewee signed a new consent form agreeing to conduct the interview and to allow its recording.

The interview guide included nine questions about the participants’ experience, what they derived from the experience, and how the simulation compared to traditional learning in a classroom. The Interview Guide’s questions were based on a literature review of simulation evaluation and debriefing. (KD and IS compiled) Three professors at Ben Gurion University, experts in leadership, simulations, and health policy, validated the questionnaire using the content validity method. This method is based on the relevancy and coherency of a framework’s elements and the degree they represent a specific goal (25). Two questions that were not clear were revised, and two new questions were added to the Interview Guide. In the second round of review, there was a consensus among the three professors regarding the suitability of the interview guide.

All students were offered face-to-face, telephone, and Zoom interview options (Zoom is a video-telephony software program), but all chose to be interviewed by phone. Each participant was given a code (interviewee number), and no identifying details of the participants were mentioned in the interview transcription or the data analysis.

### 2.4. Data analysis

#### 2.4.1. The questionnaires

The questionnaire included an open and a closed section. The answers to the open-ended questions were analyzed using content analysis according to the Hickey & Kipping approach (26). The approach required three researchers (KD, IS, ND), two of whom worked together for much of the process, and a third researcher who verified the credibility of categories and the consistency of subsequent coding. In the first stage, KD entered the response data for each question into SPSS file v.29 (IBM, Armonk, NY, United States). During this process, ideas for categories were created, which highlighted the main themes emerging from the data. In the second stage, IS and KD read the answers (18 questionnaires for each simulation, for a total of 36 questionnaires for both simulations) and reached a consensus on the categories based on the dialog of the rationale underpinning each category. In the third stage, a copy of the responses was given to ND, who developed a set of categories compared with those identified by IS and KD. Disagreements were discussed until a final set of categories was agreed upon. In the fourth stage, IS and KD allocated categories and detailed codes. In the fifth stage, ND checked the coding decisions for accuracy and reliability. In the sixth stage, IS and KD merged and reallocated the details. In the seventh stage, ND checked the decisions regarding the merging and reallocating categories. Any discrepancies were discussed until a consensus was reached.

The codes can be seen in Appendix 5. Due to the small number of participants, the data analysis is descriptive. The frequency distribution for each answer is shown by comparing the results of the two scenarios.

#### 2.4.2. The semi-structured interviews

We used assigned numbers for each interviewee to maintain confidentiality. The average time of the interview was 20 ± 4.76 min. The interviews were recorded and then transcribed by a professional transcriber. Details that could reveal the identity of the interviewees were omitted (e.g., position, specialization, etc.).

The interviews were analyzed using a thematic analysis method based on Kolb’s experiential learning model (8). A theme expresses a broad central idea that repeatedly appears in different forms of expression in the materials. The thematic analysis of the interviews was carried out in several stages according to Shkedi’s method (27). In the first stage, KD and IS read all the interviews to familiarize themselves with the data. In the next step, ideas, categories, and themes related to the research questions were identified by each reader. After the themes were agreed upon and validated, the characterizations and ideas were discussed while rereading the transcripts until the final themes were formulated with exemplar quotes. The themes were sorted and distributed according to Kolb’s experiential learning model and according to the research objectives (8). The themes and quotes were translated and documented in English at the final stage. We used a standardized codebook to ensure the validity of the translations from Hebrew to English.

### 2.5. Ethics statement

The study was approved by the ethics committee of Ben Gurion University of the Negev (approval #198–1 dated May 25, 2022). Participants gave informed consent for inclusion in the study and were informed about the procedures planned for anonymity, data protection, and privacy. A detailed explanation was provided before the simulations, and participants were given the option to opt out. The interviewees were asked to sign a consent form detailing the purpose of the study, their right to stop the interview at any stage they wished, a promise of confidentiality, and their consent to the interview was recorded. The recordings were used for transcription purposes only and then deleted.

## 3. Results

### 3.1. Sample characteristics

Eighteen students participated in the study. Table 1 illustrates the sample’s characteristics. The individual sample characteristics are shown in Appendix 4.

**Table 1:** Grouped sample characteristics (*n* = 18).

Character	Group	*N*	%
Gender	Women	10	56
	Men	8	44
Profession	Nursing	5	31
	Medicine	4	25
	Health professions	4	19
	Administration	5	25
Family status	Married	16	88
	Singles	2	12
Religion	Jews	15	88
	Muslims	3	12

### 3.2. Questionnaires

Eighteen students completed the questionnaire after each scenario (Table 1). Ten were women (56%), and eight were men (44%). Their ages ranged from 26 to 50 (average 38 ± 7.23). Four were doctors (22%), five were nurses (28%), four were health professionals (22%), and five had administrative positions (28%). Eleven had participated in simulations in the past (61%), in the army, in studies, or their workplace.

#### 3.2.1. Open-ended questions

The students were interested in fruitful dialog, cooperation and appreciated the importance of hearing different opinions. In contrast, more than half of respondents felt that the second scenario was more challenging and complex than the first scenario. Most felt they had the skills to meet the learning objectives in both scenarios (78% in the first scenario compared to 50% in the second scenario).

The students indicated that they strengthened their interpersonal communication skills and learned about the importance of listening to the opinions of others before forming their position. They strengthened their self-confidence, the ability to express a position on key topics and convince others and learned how to make compromises. Strengths they identified concerning themselves were listening to others without interrupting, assertiveness to stand up for themselves, and the ability to convince others about their opinions. They identified weaknesses mainly in the first scenario: difficulty expressing oneself, leaving their comfort zone, and rapidly raising their voices.

The participants said they enjoyed the group dynamics, joint discussion, and presentation of the dilemma from the varied perspectives of others. They least enjoyed entrenching themselves in a position and lashing out at others. Some were disturbed by the feeling of being video recorded.

When asked: “Will participating in the simulation help you deal with similar situations in the future?” 89% answered yes. The ways of influence were diverse. For example: *“It increased my confidence to stand up and convince others of my position. I felt what it was like to stand in front of senior people and express myself, it helped me understand how to behave correctly to promote interests, and I learned how to compromise and reach a consensus.”*

The distribution of answers for each question for both scenarios is shown in Appendix 5.

#### 3.2.2. Close-ended questions

The questionnaire included nine closed questions in which participants were asked to indicate their agreement with each statement on a Likert scale. The participants were positive about both scenarios. Most respondents expressed satisfaction with the conditions under which the simulations were performed, felt confident in managing the simulated conflicts, identified the issues that require leadership skills, and their leadership ability. There was a slight improvement in responding to the questions in the second scenario. The distribution of answers for each question for both scenarios is shown in Appendix 6.

### 3.3. Interviews

Sixteen of the 18 students who participated in the simulations were interviewed (89%). Of them, 9 were women (56%), 7 were men (44%). The data analysis resulted in four main themes according to Kolb’s experiential learning stages model (8): face-to-face learning versus simulation experience; differences in experience between the two scenarios; simulation as a toolbox for the continuation of personal and professional life; and, accepting the scenario as valuable for professional training and relevant to their work.

Table 2 illustrates the themes and sub-themes according to the stages of Kolb’s experiential learning model.

**Table 2:** Themes and sub-themes according to Kolb’s model.

Stages in Kolb’s experiential learning model	Themes	Sub-themes
Active experience in a new or familiar situation that requires investigation and interpretation	I. Face-to-face learning versus simulation experience	One-person show vs. co-play Theoretical versus experiential learning
Reflective observation of the situation, whether there is a match between previous understanding and experience and the situation	II. Differences in experience between the two simulation scenarios	Micro versus macro Knowledge gaps and how best to overcome them Innocence versus taking over the discussion
Building perceptions and processing information in reflective processes that lead to changing existing perceptions	III. Simulation as a toolbox for the continuation of personal and professional learning	Listening, inclusion, and integration skills Self-confidence, a sense of higher competence Meeting management, leading, striving for achievement Learning about myself versus learning about colleagues’ skills and competencies
Examination of the newly acquired insights	IV. Accepting the scenario as training for their professional work and organizational challenges	Alleviating concerns in communication with superiors and bosses The culture of discussion

#### 3.3.1. Theme I: face-to-face learning versus the simulation experience

In the first stage of the experiential learning model, an active experience in a new or familiar situation requires investigation and interpretation. Most of the BGU University degree classes are taught in a traditional face-to-face learning modality (a lecturer standing in front of a class of students). The simulations described in this study are the first experiential experience within the master’s degree. The students were assigned roles and had to deal with the situations without guidance or external intervention. In the interviews, they noted the significant differences between the two learning methods according to the following sub-themes:

##### 3.3.1.1. One-person show vs. co-play

In face-to-face learning, the lecturer determines the order of conduct in the lesson and the course. The students are more passive, compared to the simulation - in which the students are given a platform to be interactive and leading, and the instructor is more passive.

Interviewee #1 opined: *“It’s much more interesting, and you want to participate and give of yourself more. In face-to-face learning, you sit in class and fiddle with the phone, talk with friends, and fiddle with the computer. Here you were all in action, a partner in the task.”*

Interviewee #10 emphasized the importance of everyday discourse: *“I believe in the discourse, and I like these opportunities. I like unusual things. All these courses, where you learn all kinds of theories, are very specific and defined. When there is in between the possibility to add the discourse and the interactions, people Everyone brings with them their own beliefs and life experience. The opportunity in the simulation allows much more learning. I like listening to others, and if I have something to take from someone else–it’s an opportunity in my eyes.”*

##### 3.3.1.2. Theoretical versus experiential learning

Most of the face-to-face classes are theoretical, planned, and less experiential. Although the simulation is designed, the student’s behavior cannot be predicted, which makes learning an authentic experience when the student-actor reacts to each other during the scenario. The simulation experience is an opportunity to apply and practice skills that cannot be acquired theoretically in a classroom setting in front of a lecturer.

Interviewee #15 shared: *“These are exactly what I wanted, practical tools. You can sit and talk in class for hours and hours, and it does not get to you. It would be best to practice changing something inherent in you and your habits. And practice it in a safe, controlled environment - It seems much more correct to me, and it’s also what later gets you used to function in real-time. So, I think this simulation taught me much more than all the classes. Because theory is nice and important, but if you do not apply it, you have not done anything with it. I would be happy for it to be more.”*

Interviewee #10 added: *“I remember the ability of the one who led the discussion to let people talk, exchange opinions and criticize each other and finally bring things together. Stop and sort out the important details. I said, ‘Oh, this is a point I take.’ The ability to put things together. One of the participants pulled something from “under the waist,” and I told myself that you are never prepared for every situation. I looked at the girl when he said it to her. She was speechless for a moment and did not know how to react. These things can always reach us one way or another; someone can catch you “below the belt,” and you must deal with it. It was an empowering experience, an opportunity to deal with something, and from a place where you are a part, it was a good experience.”*

#### 3.3.2. Theme II: differences in the experience between the two simulation scenarios

The second stage in the experiential learning model includes reflective observations on the simulations. The students experienced two scenarios that were not built on each other. They brought up differences in their personal experiences regarding the two scenarios in their reflective observations.

Interviewee #2 shared her feelings: *“The first was like an experience, both in my role and group dynamics. It was more relaxed. There were more requests to hear the other opinions, to let the sentences finish. The second simulation had a battle atmosphere; everyone got to say what they wanted and believed their opinion should be accepted now. There I felt less desire to listen.”*

Interviewee #4 enjoyed both scenarios equally and pointed out the challenge in the second simulation: *“I enjoyed both simulations. In terms of the role, the first role was much easier on the surface. It was easier to play it than the second role, which was more demanding and complex and required rethinking this matter.”* The reasons for the differences in feelings between the simulations included three sub-themes:

##### 3.3.2.1. Micro versus macro system scenarios

The interviewees mentioned that the first scenario was written as a discussion at the local level with characters they could more easily identify with. In contrast, the second scenario simulated an ethical dilemma among senior officials at the national level, which felt less real to them.

Interviewee #10: “*The first was more convenient to operate and was something we were familiar with, material we went over and dealt with, the team members we knew, also their thoughts on the issue. The second was more challenging, issues that stand in the way of a world without solutions. Even at the level of knowing what I want to say or what I think about, it was more challenging there. But it was also more interesting because you could be in less comfortable places, but more enabling.”*

##### 3.3.2.2. Knowledge gaps and how best to overcome them

The gaps in knowledge and familiarity with the material affected the overall experience. Interviewee #14 mentioned the prior learning that helped in the first scenario, and in contrast, the challenge and the need to “get out of the box” in the second scenario: *“The first one was within the material that we studied, and then we more or less knew what we were talking. The second simulation was a bit out of the box because we got roles we had no idea how to process.”*

Interviewee #6 expressed the fear she had and overcame the situation through the support she felt from her co-workers: *“Even though they had their role, they gave a feeling: do not be afraid, we are all in the same boat. They cooperated, and that’s what helped me. It’s an experience for life - sometimes you need good people next to you that you can trust. As soon as you do not know something, you look for someone better than you at it, and that’s what I did. That’s how I bridged my gaps.”*

##### 3.3.2.3. Innocence versus taking over the discussion

Some interviewees talked about innocence in the first scenario. From a “soft landing” into a situation that was more familiar, about which they had prior knowledge and were able to enter the role relatively quickly. On the other hand, in the second scenario, an ethical dilemma was presented concerning the sensitive area of the individual’s right versus the common good, making the discourse aggressive, in which each character wanted to voice and impose their position.

For example, interviewee #11 shared: *“The first, I felt there was innocence. The second, everybody became more aggressive. You try to give a solution to a problem in half an hour, and everyone has their position, and everyone wants to win. It does not seem realistic, and it also turbocharges these matters. It introduced more emotions and more aggressiveness. They tried to forcefully take over the discussion and not listen to what others had to say. Ultimately, I learned that not every matter and issue you represent will be the only one that will pass, that you will win over everyone, and that your opinion will be accepted. Not realistic Every discussion you participate in, the topic you promote will be the one that will receive the full attention.”*

#### 3.3.3. Theme III: simulation as a toolbox for the continuation of personal and professional learning

The third stage of the model emphasizes the importance of information processing in the reflective processes that lead to changing existing perceptions and behaviors. Simulation has a wide variety of meanings and roles that allows participants to experience a predefined experience and practice different skills. The practice enabled the participants to investigate, learn, and reach conclusions about what was good (more or less) in their conduct with others.

An attempt was made to simulate complex situations of discussions in various dilemmas while trying to reach a consensus. The process of reaching an agreement is an essential skill that must be practiced to be effectively used in real-world situations. The participants noted in the interviews that the simulations equipped them with a toolbox of different skills that were divided into four sub-themes:

(i) Listening, inclusion, and integration skills

*Listening* is one of the essential tools in the ability to lead. Interviewee #8 described the importance of listening: *“I think that some of the opinions that people have around a table in one or another meeting may require listening to the end and not being fixed in your position. Sometimes a position may be mine, and I believe in it, but it is important to listen to others because they can Be that it can change a mind.”*

*Inclusion* is an important element in listening because it is possible to listen technically, and it is also crucial to include different professional opinions. *Integration* makes it possible to connect listening and inclusion and to understand what can be derived from them. Interviewee #10 defined it from her point of view in a way that explains the importance of inclusion and integration: *“I first learned to listen, which is something that challenges us sometimes. Stop for a moment and hear all sides, integrate things. On the other hand, I was very comfortable listening to and agreeing with others, and I had to remind myself to stand by the opinion I had to present here. This is also something that I will take to life, always remembering to be both. Also, in the ability to listen, in inclusion, but on the other hand, I always have to remember who I am and what I bring. I must preserve and see how it connects.”*

(ii) Self-confidence, a sense of higher competence

*Self-confidence* relates to the leader’s ability to act, make decisions, and influence others. During a simulation, the participant can learn whether he has this feature and build on it accordingly. Interviewee #2 authentically shared the tool added to her toolbox: *“It gives a sense of security that you are sitting in a forum and can express yourself. The experience strengthens future dealings with similar situations.”*

*A sense of competence* relates to and is built through successful experiences. This feeling is acquired, and the individual must experience it to know her/his capabilities. Interviewee #2 shared: *“I think experimenting with the simulations increases the ability to deal with similar scenarios. Although I do not sit with the Minister of Finance, I do sit with seniors.”*

(iii) Meeting management, leading, striving for achievement

Decision-making is required in all emergent situations. Decisions should be based on data, execution capabilities, and more. One of the ways to make an informed decision is to have a professional discussion of the data and to listen to various opinions. Interviewee #10 explained how the simulations contributed to her professional world: *“It’s something I’m not strong at, and I’ll run into it, sitting in such meetings that are more in the noise of the world. Today I’m used to doing professional things, which I’ve known for years. It’s relatively something to sit in meetings where you talk about things from a much higher level, with a broader view of the collaborations of other factors that ultimately affect important things and all kinds of practice in the field, which is new to me. For me, it was an opportunity to dive into it and be a part of something like this in the broader view.”*

Leadership in the professional world is not measured by the definition of a role but by the execution of the task and the ability to harness additional people to the task (28). This ability is not self-evident, especially in ethically complex tasks (29). Interviewee #15 shared when asked about the relationship between professional life and simulations: *“The first simulation was quite similar because I was with people who supposedly came to hear from me, and I was being led, so it felt quite similar to me. And the second one was more difficult for me because I had to deal with the fact that they were on the same level, and I had to negotiate with them. These are different strategies. It’s more difficult. You do not come and give instructions. Everyone had their agenda, and I had to deal with it and try to lead the discussion.”*

Although reaching the required achievement is significant, one must remember how one strives for achievement. Interviewee #9 shared his strategy: “*In the conduct of striving for contact or achievement, I learned that you need to pay attention to who is sitting next to you, to the personal complexity of the people next to you. In one of the simulations, my way offended one of the partners. But okay, in real life, I get to know the people better and know a little more.”*

(iv) Learning about myself versus learning about colleagues’ skills and competencies

One of the most critical tools in the toolbox required of leaders is one of self-awareness, which is routinely built through self-learning and getting to know yourself and your colleagues. The participants emphasized that a person who thinks he can act alone will not be able to promote, manage, lead, and lead for a long time. For example, interviewee #1: *“One of the girls who was with us in the group, her character and character, that’s what I thought until the simulation, was arrogant and did not treat anyone and ignored the environment and was focused on herself. That’s how it looked. After the simulation, I got to know a completely different person, shared more, and listened to different opinions. She consulted with us, and it was ‘wow, what a completely different person,’ one person in the class and someone else in the simulation.”*

Interviewee #12 confirmed: *“I learned even from the first simulation to the second. I told myself that I would give my friends more space to speak and listen to their arguments. And as soon as I heard the sides and there were smart arguments, I connected. It helped me to be more open-minded. As if to listen more, to get more opinions.”*

Interviewee #16 added: *“The simulation allowed me to look inward and outward. Looking inward is really from the situation I learned about myself, the points that are more or less difficult for me. And it’s an amazing experience to see the extraordinary abilities of other people. Verbally as well, also in terms of group dynamics, also perceptual.”*

#### 3.3.4. Theme IV: accepting the scenario as training for their professional work and organizational challenges

The fourth stage of the experiential learning model deals with examining the new insights gained when participants face similar situations in the future. Indeed, the participants compared the simulation scenarios to their daily work life. The interviewees reported they had diverse roles in the health system, hence the variety of answers they gave, each from their perspective and personal experience. The responses were divided into two sub-themes:

(i) Alleviating concerns in communication with superiors and bosses

Interviewee #14 described the opportunity made possible by the simulation to sharpen communication skills with his superiors during discussions and to voice his opinion: *“If we put together everything, we learned about what was in the simulation, in the end I took out quite a lot of tools and quite a lot of options that I did not even think I had. Also, communication with People and communication between people from completely different worlds. Because even in the simulations, we were in all the various professions in the health systems. I used to be afraid to talk to someone in a senior position. Now I feel that I can be direct and have my opinion in front of seniors.”*

(ii) The culture of discussion

When you practice a culture of discussion through simulation, you can arrive more prepared and act in a more efficient and respectful manner. Most interviewees brought up the topic of discussion culture as a key matter. Part of leadership is the ability to conduct a discussion that respects all participants and, above all, invites all opinions to be heard to help teach an informed decision. However, as in professional life and simulations, some discussions were more heated and less respectful. Interviewee #1 described her insights that everyone should be given an opportunity, including herself: *“What I take, I’m talking about the second simulation that she was a little more aggressive and more entrenched in her position, that’s when you want to say something - say it and stand your ground because, in the end, they will hear you. I mean, not because several people together told you no, so what you say is wrong and unjust. You will bend down and follow their position. You can continue to try to convince and prove. And you do not have to say things aggressively. The message can be conveyed in a pleasant way.”*

Interviewee #4 described the discussion culture and the importance of listening and reaching a consensus pleasantly. Despite the extensive experience that professionals have, other opinions can be valuable: *“I’ve been in the profession for many years. I’ve participated in all kinds of meetings and led all kinds of things. I have experience. From the simulation, I took attention. It’s important not only to speak but also to hear. That’s what we need to learn to do, including me. We often want to voice and decide. It’s important to hear each other and reach a common denominator.”*

Interviewee 5 emphasized the importance of dealing with objections: *“First of all, I experienced dealing with people who seem programmed to have an opinion contrary to mine and how to deal with it respectfully on the one hand and matter-of-factly on the other. At the level of the simulation itself, I think it was an excellent simulation.”*

Interviewee #11 mentioned the vital element of different opinions, which can be heard in a cultural discussion: *“Discussion management. These seem to me to be the main things. I do not think you can afford in real life to behave aggressively. Maybe I’m naive. I have not experienced politics or things like that.”*

## 4. Discussion

The simulations demonstrated the unique importance of using simulation to train public health students in improving their leadership competencies. The results highlighted the need for innovative learning and experiential opportunities beyond providing students with theoretical knowledge. The innovation of our findings may lie in identifying strategies for inculcating and strengthening leadership skills in practice beyond traditional classroom learning. Similar simulations in Public Health Schools have not been conducted in Israel. The literature in the field of simulations in the healthcare system is traditionally concerned with strengthening clinical skills, and communication between therapists and patients and less with leadership, leading teams, and decisions making (30). Herein lies the contribution of simulation to strengthening leadership and decision-making through innovation and creativity.

All participants agreed that realistic-simulated leadership dilemmas were a valuable learning strategy and offered a powerful process beyond that occurs in face-to-face learning. The experience, dynamics, interaction, and interpersonal communication challenged the participants and their learning more than in their face-to-face teaching. Theoretical studies are essential, but they are different from the immersive experience, in which the students are empowered to develop skills that will be used in the future in their personal and professional lives (31, 32). These findings are consistent with previous studies, which found that experiential training has a positive influence on the learning process (33–36). Simulation is considered a safe method when learning to address unpredictable situations concerning non-technical skills and thus can improve management and leadership abilities (37). Students are more likely to progress in their learning and skill acquisition through simulations (38).

The quantitative and qualitative findings suggest that the simulation’s lasting value may lie in provoking deep reflections and insights by the participants about their leadership and management skills. Most participants saw the simulations as a bridge to explore their confidence and learn to make their voices heard more effectively during discussions and how they can encourage others to speak up. The participants gained an understanding of moving from solo leadership to teamwork and the ability to respectfully listen to the opinions of others, as in Cooper et al. (12). The study also found, as noted by Gonen et al. (39), that the simulation increased the chances of effective learning with long-term assimilation. The participants reported that they gained many tools from participating in the simulations such as strengthening their self-confidence to voice their opinion and convince others, listening more effectively, making decisions under conditions of uncertainty, and more. Similar findings were found in a study that examined the effects of participating in a daily workshop in Israel based on simulations to strengthen communication skills among 42 medical students in their Psychiatry department rotation (19). The authors found that there was a significant increase in the interpersonal communication skills of the participants, as well as improvement in their self-confidence in communicating with patients. Peleg (40) described a process of implementing simulations within an interpersonal communication course among physical therapy students. The students found the scenarios relevant to their learning process and the simulation effective and realistic. They added that they experienced the simulation as a significant event that promoted learning. Chen et al. (41) found that in simulations carried out with the aim of examining coping strategies during an emergency, the simulations reflected flexibility in decision-making among emergency incident managers.

Cooper et al. (12) developed a training program to teach key concepts of teamwork and leadership among 108 managers of US healthcare organizations. The simulations helped the participants identify issues with self-confidence encouraging the students to acknowledge that they were afraid to speak their minds. Some commented on their failure as leaders to invite others to speak up. Others recognized the need to improve teamwork and communication. The participants in the current study raised similar points of weakness, and it became clear that there is a need to strengthen these skills during the socialization to the profession as part of the studies and in their workplaces.

### 4.1. Limitations

Our study has several limitations. First, we had a limited number of participants in our pilot study, and all were selected from a training course in leadership. However, the students came from various backgrounds and roles so that a multi-disciplinary team could be simulated in each simulation. Second, the study results reflect the Israeli health and education system, which may not be generalizable to other countries with their distinct health delivery and training systems, comprising unique legislative and organizational characteristics, and within diverse clinical and political settings. Third, we cannot be sure how effectively the lessons from this study are generalizable to public health leaders in real world situations. Fourth, the study did not account for the “learning style” of the participants. Fifth, the interviews were transcribed from Hebrew, the native language in Israel. This may have increased the chances for variations in the interpretation of our data. We made all efforts to ensure methodological rigor and validity of the translations from Hebrew to English by using a standardized codebook, meeting frequently, sharing and comparing our results, and performing a pilot analysis. Throughout the study, we conducted an ongoing internal quality audit during our meetings, adapted from Mays & Pope (42) and Tong et al. (43), to determine whether the data were collected, analyzed, and reported consistently according to the study protocol.

## 5. Conclusions and recommendations

The results from the study demonstrate that simulation training is a powerful pedagogical tool in the leadership education of public health leaders. Simulation realistically conveys critical issues regarding leadership and decision-making, teamwork challenges, and can instill a culture of conducting substantive, reflective, and respectful discussions. Simulation has many advantages over face-to-face traditional learning in imparting skills, feedback, and practice. The simulation allows a powerful emotional experience tailored to different professional and emergency contexts essential to developing public health leaders. We recommend integrating simulations in all public health leadership courses. Further research is needed to examine the long-term effectiveness of simulations on managing meetings, reaching consensus, persuasiveness and self-expression, and decision-making skills in times of emergency and crises. This pilot study will help to further enhance our leadership programs by providing powerful scenarios for imparting essential management and leadership skills. Future work is needed to perform an objective assessment of the participant’s performance in simulations and real-world situations.

## Data availability statement

The raw data supporting the conclusions of this article will be made available by the authors, without undue reservation.

## Ethics statement

The studies involving human participants were reviewed and approved by the ethics committee of Ben Gurion University of the Negev (approval #198–1 dated May 25, 2022). The participants provided their written informed consent to participate in this study.

## Author contributions

KD, ND, and IS: conceptualization, analysis, and interpretation. OV and IS: project administration. KD and IS: data curation. KD and PB: writing-original draft. IS, ND, OV, KC, JN, RO, LL, OB, MD, HL, FM, MM, LO-E, SZ-S, and PB: review and editing. All authors contributed to the article and approved the submitted version.

## Funding

The project is co-financed by EU funds within the framework of the Erasmus+ Program of the European Union [Grant Agreement 618578-EPP-1-2020-1-BE-EPPKA2-CBHE-JP].

## Conflict of interest

The authors declare that the research was conducted in the absence of any commercial or financial relationships that could be construed as a potential conflict of interest.

## Publisher’s note

All claims expressed in this article are solely those of the authors and do not necessarily represent those of their affiliated organizations, or those of the publisher, the editors and the reviewers. Any product that may be evaluated in this article, or claim that may be made by its manufacturer, is not guaranteed or endorsed by the publisher.

## Author disclaimer

The European Commission’s support for the production of this publication does not constitute an endorsement of the contents, which reflect the views only of the authors, and the Commission cannot be held responsible for any use which may be made of the information contained therein.
