# Supplementary material for: Simulation as a key training method for inculcating public health leadership skills: a mixed methods study
Source: Front Public Health. 2023 Jul 6;11:1202598. doi: 10.3389/fpubh.2023.1202598 (PMC10359821; doi:10.3389/fpubh.2023.1202598)
Supplement: Supplementary file 1 [file Data_Sheet_1.docx]

## **Appendix 1 - The simulations**

## Scenario 1- Leadership in a complex environment

| Leadership in a complex environment | | | Simulation title |
| --- | --- | --- | --- |
| 20 min | | | Estimated time: |
| 40 min (20 without watching the video footage) | | | Estimated debriefing time |
| Core: developing leadership skills   1. Leading a coalition of parties outside the health system to promote change 2. Creating a vision and a plan for action 3. Creating a plan for removing barriers identified by the team   Learning outcomes: students who complete the scenario will demonstrate an ability to use leadership skills to form a coalition and mobilize stakeholders from different fields to action.  Specific learning objectives:   1. To advance processes in a complex environment 2. To create collaborations with parties outside of the health system 3. To develop a vision and relay it to stakeholders 4. To define barriers 5. Planning how to overcome barriers. Thinking outside the box 6. Managing a team 7. Managing conflicts.   Prior to the simulation:  Each participant will read the scenario carefully and realize their specific role.  Each participant will sign an informed consent form for recording the session for learning purposes. | | | |
| a) Case abstract | | | |
| The vaccination rate of children in the city of Be'er Sheva aged 5-12 against COVID-19 is around 24%, whereas the national goal was set to 75%. One of the main components of the lack of adherence to children's vaccination against COVID is distrust in the health system and parental concern over side effects.  The largest HMO in the city did not designate enough facilities, flexible hours and manpower for the vaccination operation. The HMO turned to the municipality in a request to vaccinate in the community centers spread across the city, and the municipality is taking its time with responding. COVID incidence among children is rising, and the mission to increase vaccination rates is of great national importance. | | | |
| b) Main context | | | |
| The minister of health is expressing his concern to the COVID project manager given the high incidence rates and low vaccination rate in the city. The project manager convenes a multidisciplinary team for a discussion on what he views as a national responsibility, as well as on the barriers that prevent parents from vaccinating their children. | | | |
| c) Team participating in the scenario | | | |
| COVID tsar (COVID national coordinator ( | Head of the city's parental committee. | Negev regional HMO manager | |
| The mayor | Director of education |  | |
| d) The environment | | | |
| Mayor's office | | | |
| e) Required equipment | | | |
| Microphones | Cameras | A quite room | |
| A recording program | A table circled by chairs |  | |
| f) description of the participating characters | | | |
| COVID national coordinator- a doctor with a rich history in administrative roles, including hospital administration. Has been in office for about a year and is determined to prove himself so he could get to a more senior position within the ministry of health. He is assertive and extreme in his positions regarding children's vaccination. In his opinion, parents should be forced to vaccinate their kids. He is under tremendous pressure in the wake of a new wave of COVID which he is trying to prevent through encouraging vaccinations. The prime minister and minister of health are urging him to increase vaccination rates and lower incidence rates without imposing further restrictions on the population. The project manager is the one who initiated the meeting and is leading the discussion. His goal is to plan an intervention program that will incorporate all parties in order to increase vaccination rates.   \| The mayor- In his 40's, father of 2 vaccinated children. He takes the side of the project manager on the issue of raising the vaccination rates, not through coercion, but rather through soft interventions, advocacy, and persuasion. He refuses to allocate community centers as vaccination centers for the HMO. From his perspective it is a hang-out place for children, and he doesn't wish to see it turned into a medical facility. He is angry at the district manager who pressures him to authorize this. He wants to raise vaccination rates in the city, but also to look after citizens' welfare and to appease them, meaning not turning urban recreational facilities into vaccination facilities. He thinks the HMO can put in more effort and allocate its own facilities for this purpose. He also wishes to issue a press release on the planned intervention program after the meeting. \| \| --- \| \| Head of the city's parental committee- approaching age 40, an energetic high-tech manager, highly intelligent and difficult to persuade. Father of 3 three children aged 6, 9 and 12. All of whom study in the education system. He and his wife were vaccinated, but they are hesitant to vaccinate their children. He is dealing with many inquiries of parents regarding the vaccines. He is angry at the project manager for his intent to force parents to vaccinate their kids and asks pediatric doctors to explain the side effects to the parents, so as to alleviate their concerns. \| \| Director of education- 55 years old. He has a PhD in education. Father of adults. Seeks to create a climate of transparency, involvement, and partnership with all parties for the good of the children. Interested in open dialogue with the students, parents, and pedagogical staff. He believes it is important to act with sensitivity and to develop a feeling of solidarity among the parents who are concerned about vaccinating their kids. He is also interested in coming up with solutions for immune suppressant students and staff. He is against pedagogical staff giving information on the vaccines and insists that this is job of the health system. He isn't interested in vaccination knowledge translation to parents or kids on school grounds. \| \| Negev regional HMO manager- a medical doctor, has been in office for 4 years. Has experience in a variety of managerial positions in the southern district of the HMO. He is under a huge workload and is going around the different cities to try and convince people to get vaccinated. Due to the shortage of HMO facilities and the difficulty to allocate rooms for the vaccines he turned to the mayor in a request to allocate designated rooms in local community centers. The mayor refused which created tension between the two. He is angry at the mayor and is trying to convince the rest of the team members to help him get an authorization to vaccinate in community centers. \| | | | |

## **Scenario II- Decision-making and dealing with ethical dilemmas**

| Decision-making and dealing with ethical dilemmas | | Simulation Title | |
| --- | --- | --- | --- |
| 20 min | | Estimated time: | |
| 40 min (20 if not watching the video footage) | | Estimated debriefing time: | |
| Core: developing leadership skills   1. Decision-making under pressure 2. Dealing with ethical dilemmas 3. Dealing with objections   Learning outcomes: students who complete the scenario will demonstrate the ability to use leadership skills to make decisions under pressure and deal with ethical dilemmas and objections.  Concrete learning objectives:   1. Ability to make decisions (sometimes “unpopular”) under pressure 2. Dealing with objections from within and from outside of the health system 3. Discussing ethical dilemmas, advantages, disadvantages, and alternatives. 4. Managing conflicts while defending the decision and conveying it outwards through the press.   Prior to the simulation:  Each participant will read the scenario carefully and realize their specific role.  Each participant will sign an informed consent form for recording the session for learning purposes. | | | |
| a) Case abstract | | | |
| The state of Israel, like the rest of the countries in the world, is combating the corona virus. At the beginning of the month a new variant has been discovered in Africa, a deadly and contagious one. The variant has leaked to the country of Israel through Israelis who stayed in Africa, contracted the variant and brought it into the country. More Israelis, some vaccinated and some unvaccinated, were found to be carriers of the new variant. In light of the experience with the delta variant, which is also very contagious and caused the outbreak of a fourth wave (after there have been nearly no new cases in the country), there is a concern that the new variant will cause a fifth wave outbreak of corona incidence. | | | |
| b) Main context | | | |
| In light of previous experience and the concerns over a fifth wave outbreak, and due to the fact that the new variant is deadly and contagious, and it is unclear whether it is resistant to the vaccine, the minister of health has convened different parties to discuss ways of preventing further spread of the variant. | | | |
| c) Team participating in the scenario | | | |
| Minister of health | Head of public health services | | Corona National coordinator |
| Minister of finance | Minister of defense | |  |
| d) The environment | | | |
| Minister of health's office | | | |
| e) Required equipment | | | |
| A quite room | Cameras | | Microphones |
| A recording program | A table circled by chairs | |  |
| f) description of the participating characters | | | |
| Minister of health- in his 50's. has been appointed to be minister of health about half a year ago as part of the forming of a new government. He stands at the head of a radical left-wing party that advocates for guarding individual rights. It is important to him to raise vaccination rates. He encourages communication and actively advocates for the importance of the vaccines, and like any public figure he is very concerned with public relations around the issue. Thus, he is trying to produce an image of persuasion and advocacy rather than coercion. He realizes that the African variant may be lethal and highly contagious. He is supporting the stance of the head of public health services, according to which the spread of the variant must be contained so that we can try and avoid a fifth wave, which may lead to further strain on the health system and its already burned-out staff. However, since he stands at the head of a party that advocates for individual rights, he is uncomfortable with the idea of cellphone tracking of the variant's carriers by the GSS (General security service, Israel's equivalent of the FBI). The minister of health is the one who initiated the meeting and is leading the discussion. He must decide on this issue, that is between individual rights on the one hand, and guarding the public's health and keeping the health system from collapse on the other. Meanwhile it is well-known that he adamantly opposed cell-phone tracking for the same purpose when the previous government was using it. | | | |
| COVID national coordinator- a doctor with a rich history in administrative roles, including hospital administration. Has been in office for about a year and is determined to prove himself so he could get to a more senior position within the ministry of health. He is Assertive and extreme in his positions regarding children's vaccination. In his opinion, parents should be forced to vaccinate their kids. He is under tremendous pressure in the wake of a new wave of COVID, especially upon the appearance of the African variant in the country. He is an enthusiastic supporter of cell-phone tracking by the GSS in order to eradicate the plague, and from his point of view, the ends justify the means. | | | |
| Head of public health services- a doctor, in his 40's, entered office two years ago during the pandemic and is fighting to end it. He is assertive and interviews a lot for the media. He is dealing with harsh criticism from the public on his conduct, with slander and even threats to his life by anti vaxxers and Covid deniers. He is less extreme in his position than the national coordinator and is not convinced that a tool which violates individual rights should be used for the relatively few cases discovered in the country. He trusts the epidemiological investigations conducted by the healthcare system. | | | |
| Minister of finance- in his 60's. an assertive and tough type. Has been in office for 6 months. Has filled many meaningful roles during his rich political career. Has emigrated to Israel from the former Soviet Union, so government intervention in citizens' lives is nothing new to him. He is interested in avoiding sanction on the population for as long as possible, so as not to harm the economy, and therefore supports GSS cellphone tracking of carriers of the African variant. From his perspective, the good of the country is above all else, even if invasion to privacy is required by security services. He does not trust the healthcare system to perform the best investigation to identify infections. | | | |
| Minister of defense- in his 60's. In the past he assumed senior commanding positions in the military. He has been in office for 2 years as minister of defense. Not an assertive type. As part of his role, he is responsible for the secret security services, and was therefore invited to the meeting. He is not sure that the extreme measure of cellphone tracking should be taken, but he is not intending to oppose whatever is decided. | | | |

## **Appendix 2 - Questionnaire**

| Demographic questions |
| --- |
| 1. What is your position at work? 2. What was your role in the simulation? 3. How old are you? 4. Have you participated in simulations before? If so, please state when, where, and what the goal of the simulation was. |
| Open-ended questions |
| 1. How did the experience feel to you? 2. Did you have the knowledge and abilities to achieve the scenario's learning objectives? 3. What relevant information was missing in the scenario that affected your performance? 4. How did you overcome the knowledge gap? 5. What did you take from the simulation experience? 6. What strengths or weaknesses of yours were you able to identify? 7. What did you enjoy the most in the simulation? 8. What did you enjoy the least in the simulation? 9. Will participation in the simulation help you when encountering similar situations in the future? |
| Closed questions (Likert Scale, 1-5 (1=from not at all; 5= very much) |
| 1. How satisfied were you with the conditions under which the simulation was performed? (Room, equipment, etc.) 2. How confident did you feel having the discussion? 3. How confident did you feel in identifying issues requiring leadership and your own leadership ability? 4. To what extent did you feel that the discussion was run in a respectful manner? 5. To what extent did you feel you were furthering the team's dynamics? 6. To what extent did you feel able to influence others? 7. To what extent did you feel you could have been a role model in your professional behavior? 8. To what extent could you defend the decision and draft a press release? 9. How satisfied were you with the results and the conveyed message? |

## **Appendix 3 - Interview Guide**

1. Tell me a little about yourself, your age, marital status, workplace, position, and why you came to study in the leadership track?
2. Did you feel comfortable during the simulation? Please specify.
3. Which simulation did you enjoy more (the first or the second), and why?
4. What gaps have you identified in your knowledge or preparation for the simulation? How did you overcome the gap?
5. What did you take from experience in the simulation to your professional or personal world? Please specify.
6. How would you define the experience compared to traditional learning in the classroom? Is there any added value to the simulation? Should more simulations be integrated into the curriculum?
7. Please indicate a significant experience that you had in a simulation. (Something you learned about yourself or your classmates)
8. As a future leader in the healthcare system, how much did the simulation contribute to building your values/path?
9. Would you like to add anything?

## **Appendix 4 - Individual characteristics of the Sample**

**Table 3: Individual characteristics of the Sample**

| **Profession** | **Family status** | **Age** | **Sex** | **No** |
| --- | --- | --- | --- | --- |
| Administrative manager in the community | Married+4 | 39 | female | 1 |
| Nurse in the community | Married+2 | 33 | female | 2 |
| Nurse at hospital | Married+2 | 30 | female | 3 |
| Nurse at hospital | Married+3 | 46 | male | 4 |
| Paramedic | Married | 30 | male | 5 |
| Secretary at the South Health District | Married+2 | 41 | female | 6 |
| Physician at hospital | Married+1 | 30 | male | 7 |
| Physician at hospital | Single | 26 | male | 8 |
| Engineer | Married+3 | 50 | male | 9 |
| Nurse at hospital | Married+3 | 33 | female | 10 |
| Physician at hospital | Married+4 | 48 | male | 11 |
| Office manager at a hospital | Married+3 | 33 | female | 12 |
| Physiotherapist at hospital | Married+4 | 45 | female | 13 |
| Nurse at hospital | Married+1 | 47 | male | 14 |
| Physician in hospital | Single | 39 | female | 15 |
| Speech therapist in the community | Married+6 | 38 | female | 16 |
| Office manager at a hospital | Married+4 | 35 | female | 17* |
| Speech therapist in the community | Married+2 | 44 | male | 18* |

*Subjects 17 and 18 were not interviewed

## **Appendix 5 - Distribution of answers to open-ended questions**

Table 4 shows the distribution of the coded answers to the open questions in each scenario.

**Table 4:** **Distribution of answers to open-ended questions***

| **Question** | **Answers** | **Scenario I** | | **Scenario II** | | |
| --- | --- | --- | --- | --- | --- | --- |
|  |  | **n** | **%** | | **n** | **%** |
| How did the experience feel to you? | New way of thinking and ideas/different opinions | 5 | 20 | | 2 | 10 |
|  | Interesting, empowering, and educational | 5 | 20 | | 3 | 18 |
|  | Pleasant and respectful atmosphere | 5 | 20 | | 2 | 10 |
|  | Challenging and complex | 1 | 4 | | 10 | 52 |
|  | Fruitful conversation and a sense of cooperation | 5 | 20 | | 2 | 10 |
|  | Realistic | 1 | 4 | | - | - |
|  | At first, it's awkward, and later, we got down to business | 3 | 12 | | - | - |
| Did you have the skills to achieve the learning objectives? | Slightly | 3 | 17 | | 5 | 28 |
|  | Moderately | 1 | 6 | | 4 | 22 |
|  | very much | 14 | 78 | | 9 | 50 |
| Was information missing in the scenario that affected your performance? | Administrative data | 4 | 30 | | - | - |
|  | Does the vaccine help, and how dangerous is the situation? | 1 | 8 | | 4 | 24 |
|  | Satisfaction of the population with the moves | - | - | | 1 | 6 |
|  | Did the move prove itself? | - | - | | 5 | 30 |
|  | The interests and positions of the officials | 6 | 46 | | 3 | 17 |
|  | The powers of the incumbent | - | - | | 3 | 17 |
|  | How flexible can I be in my position | 2 | 16 | | 1 | 6 |
| How did you overcome the knowledge gap? | Consultation with the friends in the simulation | 2 | 17 | | 3 | 17 |
|  | Improvisation | 2 | 17 | | 9 | 50 |
|  | Dialogue while performing and understanding the different positions | 8 | 67 | | 12 | 67 |
| What did you take away from the simulation experience? | I discovered skills I didn't think I had - imagination and creative thinking | 2 | 11 | | 1 | 6 |
|  | Interpersonal communication/listening to others before forming a position | 6 | 34 | | 4 | 27 |
|  | know how to compromise and bridge | 2 | 11 | | 2 | 14 |
|  | You could see a role with a different eye from a new place | - | - | | 4 | 27 |
|  | Empathy/not hurting others | - | - | | 1 | 6 |
|  | Management of discussion and negotiations in a short time | - | - | | 1 | 6 |
|  | Ability to express a position and convince people from different fields | 3 | 17 | | - | - |
|  | new knowledge | 1 | 5 | | - | - |
|  | Self confidence | 3 | 17 | | 2 | 14 |
|  | Managing different parties with different desires | 1 | 5 | | - | - |
| What strengths were you able to identify? | Speech fluency, acting, and flow | 4 | 28 | | 2 | 12 |
|  | Listening to others without interfering | 2 | 14 | | 5 | 32 |
|  | You were able to get out of your comfort zone and talk | 2 | 14 | | 1 | 6 |
|  | Don't get carried away on personal lines | - | - | | 1 | 6 |
|  | Managing negotiations and wanting to solve the problem as quickly as possible | 1 | 7 | | 1 | 6 |
|  | Assertiveness, standing up for myself, and trying to convince | 4 | 28 | | 5 | 31 |
|  | Thinking outside the box | - | - | | 1 | 6 |
|  | Team management | 1 | 7 | | - | - |
| What weaknesses were you able to identify? | Difficulty expressing myself | 3 | 33 | | 1 | 25 |
|  | Inability to react quickly and "pull from the hip" | - | - | | 1 | 25 |
|  | Lack of confidence | 1 | 11 | | - | - |
|  | Difficulty getting out of the comfort zone | 2 | 22 | | - | - |
|  | Aggressiveness, rapid rise to a high tone | 2 | 22 | | - | - |
|  | Difficulty dealing with lack of information in real-time | - | - | | 1 | 25 |
|  | Lack of persuasion | 1 | 11 | | 1 | 25 |
| What did you enjoy the most about the simulation? | The creative ideas and solutions | 2 | 11 | | 1 | 6 |
|  | The group dynamics/joint discussion | 13 | 72 | | 11 | 65 |
|  | Hear the dilemma from each participant's point of view | 3 | 17 | | 4 | 23 |
|  | The authenticity | - | - | | 1 | 6 |
| What did you enjoy the least about the simulation? | Complex disagreements/fortification in a position/ dead end | 2 | 25 | | 3 | 33 |
|  | From the feeling of being filmed | 4 | 50 | | - | - |
|  | From the lack of knowledge | - | - | | 3 | 33 |
|  | To burst into others' sentence/raising voice | 2 | 25 | | 3 | 33 |
| Will participation in the simulation help you when encountering similar situations in the future? | I hope so | 2 | 11 | | 2 | 11 |
|  | Yes, I felt what it was like to stand in front of senior people and express myself | 3 | 17 | | 1 | 6 |
|  | Yes, the listening, the integration and the ability to look at a problem "from above" | 1 | 6 | | 1 | 6 |
|  | Yes, helped me understand how to behave correctly to promote interests | 2 | 11 | | 4 | 22 |
|  | Yes, I have identified my strengths and weaknesses, and this will help improve them | 2 | 11 | | 2 | 11 |
|  | Yes, it increased my confidence to stand up for myself and convince others of my position | 4 | 22 | | 6 | 33 |
|  | Yes, I learned how to manage a group with different desires | 1 | 6 | | 1 | 6 |
|  | Yes, I learned how to compromise and reach a consensus | 3 | 17 | | 1 | 6 |

* The participants could indicate several answers. Therefore, the total number of responses can exceed the number of participants.

## **Appendix 6- Distribution of responses to the closed-ended questions**

To present the findings, we grouped the responses into three categories: 1+2- to a small extent, 3- to a moderate extent, 4+5- to a large extent.

**Table 5: Distribution of responses to the closed-ended questions**

| **Scenario II** | | **Scenario I** | | **Answers** | **Questions** |
| --- | --- | --- | --- | --- | --- |
| **%** | **N** | **%** | **n** |  |  |
| - | - | - | - | to a small extent | How satisfied were you with the conditions under which the simulation was performed? |
| 6 | 1 | 6 | 1 | to a moderate extent |  |
| 94 | 17 | 94 | 17 | to a large extent |  |
| - | - | - | - | to a small extent | How confident did you feel talking during the discussion? |
| 11 | 2 | 11 | 2 | to a moderate extent |  |
| 89 | 16 | 89 | 16 | to a large extent |  |
| - | - | 6 | 1 | to a small extent | How confident did you feel identifying issues requiring leadership and your own leadership abilities? |
| - | - | 12 | 2 | to a moderate extent |  |
| 100 | 17 | 82 | 14 | to a large extent |  |
| - | - | - | - | to a small extent | To what extent did you feel that the discussion was led in a respectful manner? |
| - | - | 6 | 1 | to a moderate extent |  |
| 100 | 18 | 94 | 17 | to a large extent |  |
| - | - | 6 | 1 | to a small extent | To what extent did you feel you furthered the team's dynamics? |
| 12 | 2 | 19 | 3 | to a moderate extent |  |
| 88 | 14 | 75 | 12 | to a large extent |  |
| - | - | 6 | 1 | to a small extent | To what extent did you feel you were able to influence others? |
| 25 | 4 | 12 | 2 | to a moderate extent |  |
| 75 | 12 | 82 | 13 | to a large extent |  |
| - | - | 6 | 1 | to a small extent | To what extent did you feel you were a role model in your professional behavior? |
| 18 | 3 | 12 | 2 | to a moderate extent |  |
| 82 | 14 | 82 | 13 | to a large extent |  |
| - | - | - | - | to a small extent | To what extent could you defend the decisions and draft a press release? |
| 12 | 2 | 17 | 3 | to a moderate extent |  |
| 88 | 15 | 83 | 15 | to a large extent |  |
| - | - | - | - | to a small extent | How satisfied were you with the results and the key messages? |
| 6 | 1 | 11 | 2 | to a moderate extent |  |
| 94 | 17 | 89 | 16 | to a large extent |  |
